# A Myristoyl-Binding Site in the SH3 Domain Modulates c-Src Membrane Anchoring

**DOI:** 10.1016/j.isci.2019.01.010

**Published:** 2019-01-14

**Authors:** Anabel-Lise Le Roux, Irrem-Laareb Mohammad, Borja Mateos, Miguel Arbesú, Margarida Gairí, Farman Ali Khan, João M.C. Teixeira, Miquel Pons

**Affiliations:** 1 BioNMR Laboratory, Inorganic and Organic Chemistry Department, Universitat de Barcelona, Baldiri Reixac, 10-12, 08028 Barcelona, Spain; 2 NMR Facility, Scientific and Technological Centers, Universitat de Barcelona, Baldiri Reixac, 10-12, 08028 Barcelona, Spain; 3 Department of Biochemistry, Abdul Wali Khan University, Mardan 23200, Pakistan

**Keywords:** Structural Biology, Protein Structure Aspects, Biophysics

## Abstract

The c-Src oncogene is anchored to the cytoplasmic membrane through its N-terminal myristoylated SH4 domain. This domain is part of an intramolecular fuzzy complex with the SH3 and Unique domains. Here we show that the N-terminal myristoyl group binds to the SH3 domain in the proximity of the RT loop, when Src is not anchored to a lipid membrane. Residues in the so-called Unique Lipid Binding Region modulate this interaction. In the presence of lipids, the myristoyl group is released from the SH3 domain and inserts into the lipid membrane. The fuzzy complex with the SH4 and Unique domains is retained in the membrane-bound form, placing the SH3 domain close to the membrane surface and restricting its orientation. The apparent affinity of myristoylated proteins containing the SH4, Unique, and SH3 domains is modulated by these intramolecular interactions, suggesting a mechanism linking c-Src activation and membrane anchoring.

## Highlights

• The myristoyl group of c-Src interacts with the SH3 domain
• The Unique Lipid Binding Region modulates myristoyl-related interactions
• Mutations in the Unique and SH3 domains affect membrane anchoring
• A fuzzy intramolecular complex is retained in the lipid-anchored form of USH3 Src

## Graphical Abstract

**Figure:**
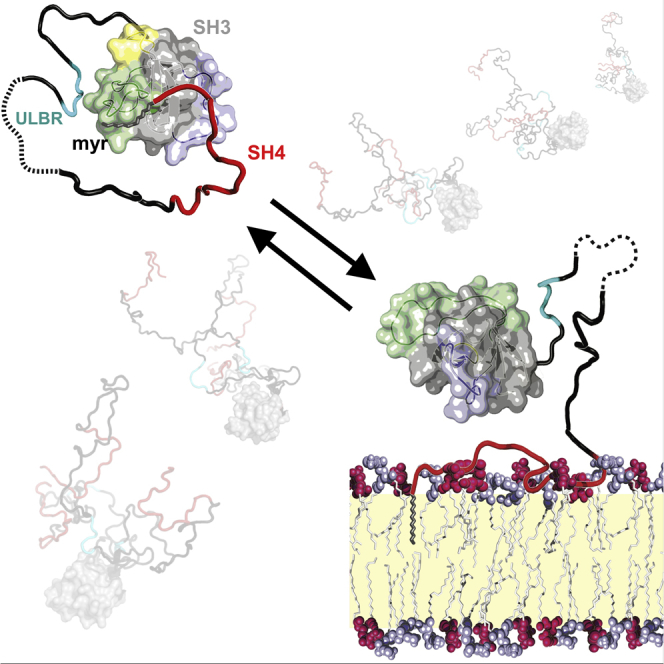

## Introduction

c-Src is the leading member of the Src family of kinases (SFK). Its oncogenic potential was brought to light already in 1970 (Duesberg and Vogt, 1970). Since then, c-Src has been associated to a plethora of cell signaling pathways and has emerged as a key player in the regulation of cell adhesion, growth, movement, differentiation, and therefore cell invasion and survival. c-Src deregulation is directly associated to poor prognosis in colorectal and breast cancer (Martin, 2001, Yeatman, 2004; Sen and Johnson, 2011, Sirvent et al., 2012). c-Src shares with the other SFKs a common domain arrangement formed by the membrane-anchoring SH4 region followed by the Unique domain (UD), and the SH3, SH2, and kinase domains. The SH3, SH2, and kinase domains can adopt a closed, autoinhibited form stabilized by interactions between the SH2 domain and a phosphotyrosine residue near the C terminus, as well as additional interactions involving the SH3 domain (Xu et al., 1999).

Importantly, membrane binding is essential for the transforming activity of v-Src and for the activation of c-Src by a membrane-bound phosphatase (Kamps et al., 1986, Bagrodia et al., 1993). All SFKs are myristoylated at the N terminus of the SH4 domain (Resh, 1994). A second lipid interaction motif is required for effective membrane anchoring. This is provided by palmitoylation of cysteine residues in most SFKs and by electrostatic interaction of the positively charged SH4 domain with the negatively charged lipids in the case of c-Src (Murray et al., 1998).

The UD is intrinsically disordered and the most divergent region in the SFK. Its role remains poorly understood. Recently, the interplay between the UD and SH3 domain has been deciphered, in which the SH3 domain acts as a scaffold of a fuzzy complex that includes the UD and SH4 domain (Maffei et al., 2015, Arbesú et al., 2017). Moreover, additional lipid-binding regions were identified in the UD and the SH3 domain by nuclear magnetic resonance (NMR) titrations with lipid bicelles in non-myristoylated constructs (Pérez et al., 2013). The UD residues affected by lipid binding included S51, A53, A55, and the 60–67 region, which we refer to as the Unique Lipid Binding Region (ULBR). Replacing residues 63–65 (Leu-Phe-Gly) by alanine (AAA mutant) abolished lipid binding by this region. Mutation of the same residues in the context of the full-length myristoylated c-Src highlighted the critical role of the ULBR because it resulted in a 50% reduction of the invasive capacity of c-Src-dependent human colorectal cells (Arbesú et al., 2017), but the actual mechanism still needs to be described.

On the other hand, the subcellular location of c-Src critically affects its function (Dwyer et al., 2016), and c-Src localization and trafficking are not fully understood. c-Src can be found at the plasma, perinuclear, and endosomal membranes (Konitsiotis et al., 2017), and also in the cytoplasm (Donepudi and Resh, 2008) and nucleus (Honda et al., 2016). Endosomal recycling has been found to be crucial for the maintenance of c-Src enrichment at the plasma membrane (Konitsiotis et al., 2017). Trafficking of c-Src between these different compartments lacks a comprehensive description.

Here we used NMR, very well suited for the study of proteins containing disordered domains in solution, and surface plasmon resonance (SPR) to characterize the myristoylated N-terminal region of c-Src, including the SH4 domain, UD, and SH3 domain, in solution and its binding to liposomes. We found that the N-terminal myristoyl group binds to the SH3 domain (in the so-called RT loop) in free c-Src. This provides an additional stabilizing element to the previously described intramolecular fuzzy complex in which the folded SH3 domain acts as a scaffold for the intrinsically disordered regions. In the presence of liposomes or supported lipid bilayers (SLBs) the myristoyl group is released to allow anchoring to the lipid bilayer, but the interaction of the SH4 and SH3 domains and the fuzzy intramolecular complex is retained. Mutations in the UD and SH3 domain affect lipid binding by the myristoylated SH4 domain suggesting a competitive model, in which the availability or exposure of the myristoyl group is modulated by interactions involving these domains.

## Results

### The N-Terminal Myristoyl Group Interacts with the SH3 Domain in the Absence of Lipids

**Figure 1:**
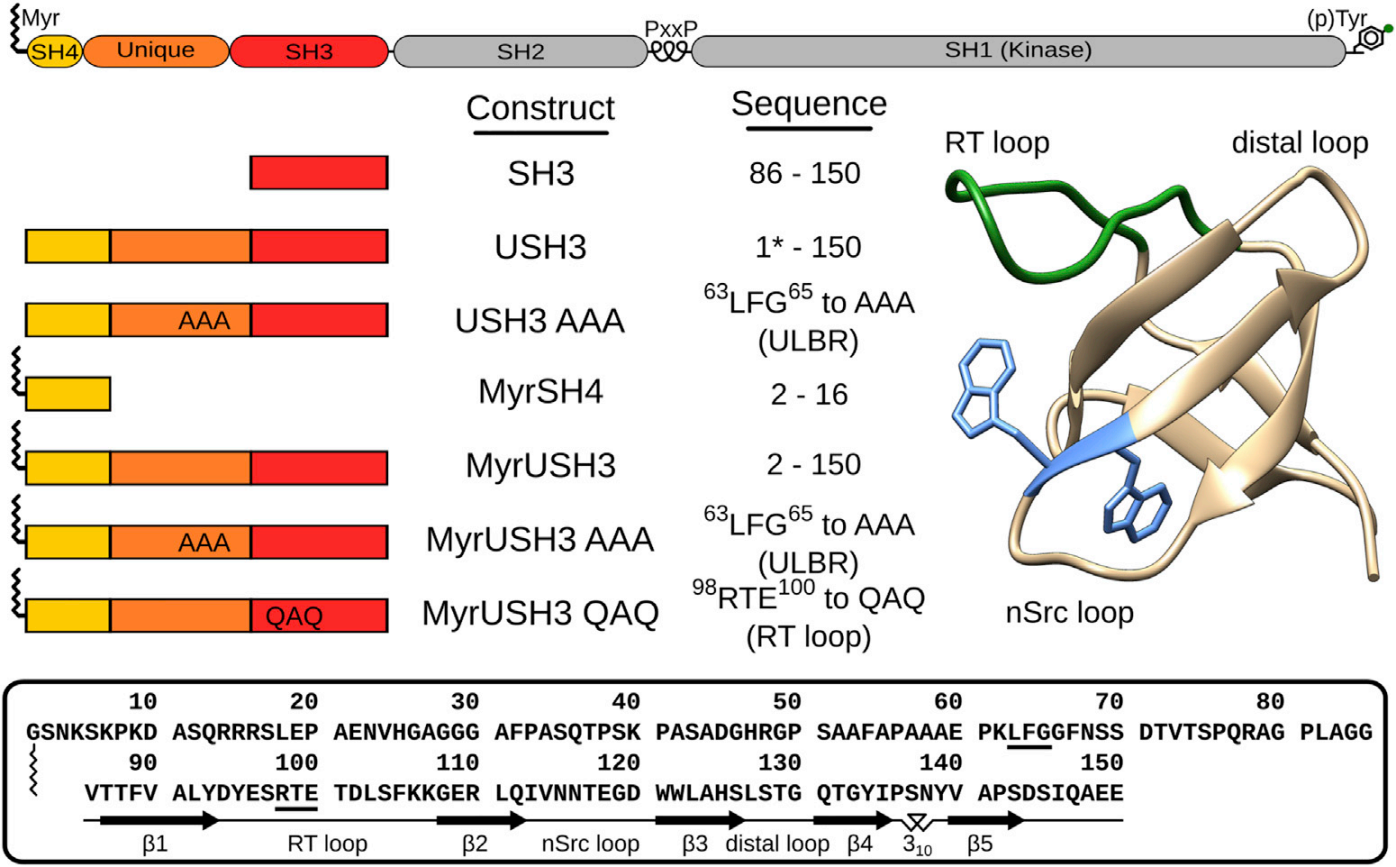
System Overview The constructs used in the study are schematically indicated. The wavy line refers to the myristoyl group attached to the N terminus. The structure of the SH3 domain, with the three loops and the two tryptophan residues, is shown in the right. The domain structure of the c-Src protein is shown on the top, and the sequence of the wild-type MyrUSH3 is indicated at the bottom. The myristoylated constructs contained a His_6_ tag after the SH3 domain. The non-myristoylated constructs contained an additional GAMA tetrapeptide that arises from cloning instead of the N-terminal glycine.

The 2–150 region of human c-Src (hereafter USH3, see Figure 1) contains the disordered SH4 domain and UD constrained around the folded SH3 domain, while retaining a high flexibility. This arrangement has been described as an intramolecular fuzzy complex (Arbesú et al., 2017). Myristoylated USH3 (MyrUSH3) was prepared in *E. coli* by co-expression with yeast N-myristoyltransferase following a previously described protocol, ensuring full myristoylation and the absence of spurious lauroylation of USH3 (Flamm et al., 2016). Using samples containing fully myristoylated proteins is crucial for *in vitro* biophysical characterization; therefore the protocol used in this study contains modifications coming from continuous improvement of the expression and purification methods. Liquid chromatography and mass spectrometry analysis confirmed that the purified proteins were 100% myristoylated (see Figure S1). Chemical shift perturbations (CSPs), calculated by comparing NMR peak positions in spectra obtained from two constructs or the same construct measured in two conditions, were used to map the residues affected by the presence of the myristoyl group or its interaction with lipid membranes. The myristoylated proteins were compared with the non-myristoylated variants or the isolated SH3 domain. The liposome-bound myristoylated proteins were compared with the same constructs measured in the absence of lipids. A schematic representation of the protein constructs used in this study, the sequence of USH3, and a three-dimensional structure of the SH3 domain are presented in Figure 1.

**Figure 2:**
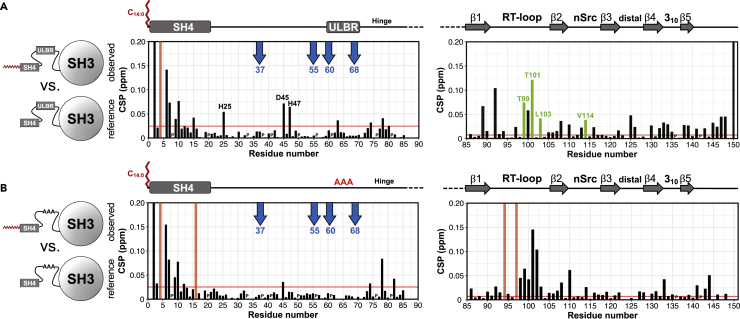
Myristoylation-Induced Chemical Shift Perturbations (A) Combined chemical shift perturbations between myristoylated and non-myristoylated USH3 WT. (Left) The SH4-Unique disordered region at 278 K. Blue arrows mark positions known to be sensitive to the formation of the fuzzy complex. (Right) The SH3 domain at 298 K. Duplicated signals are shown in green. The CSP corresponds to the highest value in each duplicated signal. Red bars mark residues that are absent only in one of the conditions compared. Unassigned residues and prolines (P) are arbitrarily given a CSP of zero. The horizontal red line represents the mean value plus five standard deviations of the 10% lowest CSP. (B) The same as (A) but for myristoylated and non-myristoylated USH3 AAA.

The CSPs of the NH group NMR signals in MyrUSH3 with respect to USH3, presented in Figure 2A, provide information on the regions most affected by the presence of the myristoyl group. SH4 domain and UD signals (Figure 2A left) were measured at 278 K, to minimize exchange with water protons. SH3 domain spectra (Figure 2A right) were measured at 298 K (also see Figure S2).

Large CSPs are found in the proximity of the RT loop of the SH3 domain, as can be visualized in Figure 2A (right). Interestingly, duplicated signals (marked in green) were observed for some of the most strongly affected residues in the SH3 domain (T99, T101, L103, and V114) in MyrUSH3. The intensities of the two peaks were close to 1:1 (see Figure S3A). As sample purity was carefully assessed, duplicated signals probably originate from slow exchange between alternative conformations.

As expected, high CSPs between the myristoylated and non-myristoylated forms are observed in the SH4 domain where the myristoyl group is attached (Figure 2A left). Apart from these perturbations, minor effects were observed in the UD, including moderate CSPs in H25, D45, and H47. Small CSPs could also be noted in L63, part of the ULBR, and in the region T74 to G80 located in the hinge connecting the UD and SH3 domain. The histidine chemical shifts are very sensitive to changes in their electrostatic environment and do not necessarily reflect direct interaction sites. Chemical shifts of key residues in the UD, namely, T37, A55, E60, K62, and N68, are diagnostic of the interaction between the UD and SH3 domain (Maffei et al., 2015, Arbesú et al., 2017), highlighted by blue arrows in Figure 2A. The extremely low CSPs observed for these key residues indicated very similar environments in the presence or absence of myristoylation confirming that the intramolecular fuzzy complex is retained in the presence of the myristoyl group.

**Figure 3:**
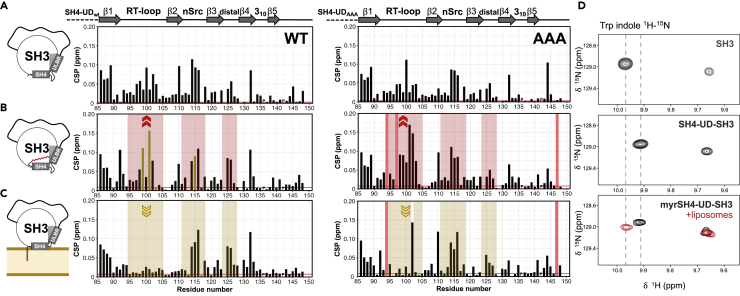
Chemical Shift Perturbations with Respect to the Isolated SH3 Domain (A) Perturbations induced by the presence of the SH4 domain and UD in the SH3 domain in USH3 WT (left) and USH3 AAA (right). (B) Perturbations induced in the SH3 domain by the presence of the myristoylated N-terminal region in USH3 WT (left) and USH3 AAA (right). The three SH3 loops are shadowed. The mark in the RT loop region highlights the increased perturbations with respect to the non-myristoylated form. Green bars correspond to duplicated signals. Red bars correspond to missing signals. (C) Perturbations induced by binding of MyrUSH3 WT (left) or Myr USH3 AAA (right) to negatively charged liposomes. The mark in the RT loop region highlights the loss of perturbations with respect to the same constructs in the absence of liposomes. (D) The indole NHs experience similar environments in free SH3 and in the liposome-bound form of myristoylated USH3. However, in the absence of liposomes, Trp 121 is sensing the presence of the disordered SH4-UD regions and the chemical shift of its indole NH does not change upon myristoylation.

Next, we used the isolated SH3 as a reference to study the effect of the presence of the myristoyl group in the preexisting interactions of the SH3 domain with the UD and SH4 domain. CSPs in Figure 3A identify the SH3 residues affected by the presence of the disordered region when it is not myristoylated (USH3), whereas Figure 3B shows the effects observed when the N terminus is myristoylated (MyrUSH3). In each panel, the wild-type (WT) and AAA mutants are compared. Large CSPs were observed in the RT and n-Src loops, but the perturbations are not the same in USH3 and MyrUSH3. The perturbation of the RT loop is larger in the myristoylated form, indicating that the myristoyl group is interacting in the proximity of this loop. A number of hydrophobic residues are located in a groove close to the RT loop (W121, W122, L123, and V140). The chemical shifts of the NH signals of these residues are not specially affected by the presence of the myristoyl group, whereas their side chains may contribute to the observed interaction of the myristoyl group with the SH3 domain.

### Insertion of Myristoyl Group in the Lipid Bilayer Competes with Its Intramolecular Interaction with the SH3 Domain

We next measured the NMR spectra of MyrUSH3 in the presence of negatively charged large unilamellar vesicles (LUVs) formed by 1,2-dioleoyl-sn-glycero-3-phosphocholine (DOPC) and 1,2-dioleoyl-sn-glycero-3-phospho-1′-rac-glycerol (DOPG) in a DOPC:DOPG ratio of 3:1. The protein concentration was 75 μM, and the total lipid concentration was 5 mM. We used the polyunsaturated lipids to ensure that lipid bilayers were in the liquid crystalline state in which proteins and lipids can freely diffuse, even at 278 K.

In the presence of liposomes, the RT loop residues displayed chemical shifts typical of free SH3, indicating that the interaction of the disordered regions of c-Src with the RT loop was lost (Figure 3C left). The indole NH NMR signal of tryptophan 121 side chains in the SH3 domain (Figure 3D) had different chemical shifts in free SH3 and in the non-myristoylated USH3. Myristoylation did not affect signal position, but when MyrUSH3 was bound to lipids, the indole NH signal recovered the chemical shifts found in free SH3.

These chemical shifts of RT loop residues are compatible with the release of the myristoyl group from the RT loop region of the SH3 domain upon insertion into the lipid membrane. The changes in indole NH signals are also compatible with the RT loop losing its interactions when the myristoyl group inserts into the lipid bilayer, as the side chain of tryptophan 121 is pointing toward the RT loop (see Figure 1).

The insertion of the myristoyl group in lipid membranes had been previously demonstrated using ^2^H-NMR in Myr-Src(2–19) (Scheidt and Huster, 2009). Importantly, the interactions in other SH3 regions, notably the n-Src loop and some residues in the distal loop were retained in the membrane-bound form (compare Figure 3C with Figures 3B and 3A).

**Figure 4:**
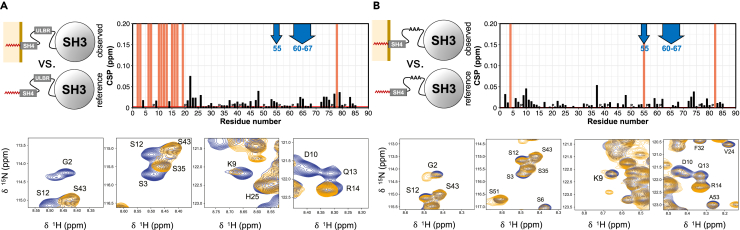
Chemical Shift Perturbations Induced by Liposome Binding (A and B) (A) In MyrUSH3 WT and (B) in MyrUSH3 AAA. The blue arrows indicate residues forming the Unique Lipid Binding Region, which is not perturbed in these experiments. Red bars mark residues that are absent only in one of the conditions compared. Unassigned residues and prolines (P) are arbitrarily given a CSP of zero. Expansions of selected regions of the SH4 domain spectra in the absence of lipids (blue) or in the presence of liposomes (gold) are presented below each of the CSP plots.

Figure 4A compares the chemical shifts of MyrUSH3 SH4 domain and UD in the presence and absence of LUVs.

As expected, the SH4 domain (positively charged) is strongly perturbed by the interaction of MyrUSH3 with negatively charged LUVs, and most signals are broadened beyond detection (Figure 4A lower panels). However, the UD shows very small CSPs. In particular, residues T37, A55, E60, and N68, previously shown to be reporters of the intramolecular fuzzy complex, were almost unperturbed in the presence of LUVs (Figure 4A). This confirms that the fuzzy complex is retained when c-Src is anchored to lipid bilayers.

Interestingly, the ULBR, which was named as such because of the interaction with lipid bicelles observed in non-myristoylated USH3, showed no chemical shift changes, in spite of the fact that the myristoylated SH4 domain of the same molecule was anchored to DOPC:DOPG 3:1 liposomes in MyrUSH3 (Figure 4A).

If the ULBR does not provide an additional interaction with the membrane lipids, the question that arises is what is its natural “lipid” target. To answer this question, we compared the effect of myristoylation of WT USH3 with that of USH3 AAA, in which the ULBR is inactive.

### The ULBR Contributes to the Interaction of the Myristoyl Group with the SH3 Domain

The assignment of the USH3 AAA construct was carried out in a ^13^C, ^15^N uniformly labeled myrUSH3 AAA sample using the targeted acquisition strategy (Jaravine and Orekhov, 2006) based on co-processing of HNCO, HNcaCO, HNCA, HNcoCA, HNCACB, and HNcoCACB spectra acquired using non-uniform sampling in the NMR facility of the University of Goteborg (Sweden). The backbone chemical shifts were used to calculate the neighbor-corrected structural propensity by comparison with a curated IDP chemical shift database (Tamiola and Mulder, 2012) (see Figure S4). The introduction of the AAA mutation does not induce a significant structure in USH3 AAA, suggesting that the observed effects result from the inactivation of the ULBR and not from additional structuring of the AAA region.

Figure 2B shows the CSP between the myristoylated and non-myristoylated forms of USH3 AAA in the N-terminal (left panel) and SH3 (right panel) regions. As observed for native USH3, the largest CSPs outside the SH4 domain occur at the RT loop of the SH3 domain. However, the AAA mutation alters the way the myristoyl group interacts with the SH3 domain, causing a distinct pattern of CSPs: residues F89 and L92 in the β1 strand were perturbed in the WT form but not in the AAA variant. In contrast, D102 was more affected in the AAA variant than in WT USH3. Residues D94 and S97, part of the RT loop, were observable in MyrUSH3 WT but were lost in the myristoylated AAA form. The duplicated signals observed for some of the perturbed residues in MyrUSH3 were lost in MyrUSH3 AAA (Figure S3B).

The changes in the SH4 domain induced by the presence of the myristoyl group are very similar in MyrUSH3 AAA and MyrUSH3, suggesting that the observed effects arise from modulation of the direct interaction of the myristoyl group with the SH3 domain, rather than from changes in the interaction of the SH4 domain.

The native and AAA USH3 variants showed similar, but not identical, effects in the UD upon myristoylation (Figures 2A and 2B left panels). H25 and H47 chemical shifts were not affected by myristoylation in the AAA variant, and D45 showed a smaller effect. The hinge region residues (74–80) were similarly affected in the AAA and native variants.

The NMR data on MyrUSH3 AAA show that although the myristoyl group interacts with the SH3 domain, changes in the UD modulate this interaction resulting in different affected residues and the absence of duplicated signals, suggesting changes in the exchange rates between alternative configurations of the fuzzy complex in the myristoylated protein.

Using the isolated SH3 domain as a common reference, CSP of the AAA variants shown in Figures 3A–3C (right panels) confirmed that the interactions with the RT loop are the most affected by the presence of the myristoyl group in the absence of lipids and mostly disappear in the liposome-bound form, similar to the observed effect in WT USH3. However, a large CSP is observed in residue D102 of MyrUSH3 AAA in the presence of LUVs, but not in WT MyrUSH3. Additional differences were observed in the distal loop showing smaller perturbations in the AAA mutant than in WT USH3 in the presence of liposomes.

Addition of LUVs to MyrUSH3 AAA caused only limited effects in the SH4 domain (Figure 4B), in contrast to the extensive broadening or large shifts observed in MyrUSH3 (Figure 4A). In contrast, in the UD, addition of LUVs resulted in the disappearance of residue A55 and a high CSP in T37, both of them involved in the fuzzy complex with the SH3 domain. Thus a native ULBR contributes to preserve the fuzzy complex when c-Src is bound to lipid bilayers.

The NMR results suggest that the AAA mutation affects the interaction of myristoylated c-Src with lipid bilayers: a reduction of the interaction of the myristoylated SH4 domain with the SH3 domain in solution facilitates membrane anchoring, and the modified CSP pattern suggests that the SH4-anchoring group is presented to the membrane differently by the retained intramolecular complex involving the SH3 domain and the WT or AAA-modified UD.

### The Unique and SH3 Domains Modulate Lipid Binding by the Myristoylated SH4 Domain

To assess the effect of the UD and SH3 domain we compared the binding of MyrUSH3 to SLBs with that of the isolated myristoylated SH4 domain (MyrGSNKSKPKDASQRRR noted MyrSH4). The role of ULBR in the UD was tested using MyrUSH3 AAA. The importance of the RT loop in the SH3 domain was tested using a mutant USH3 domain with key residues in the RT loop ^98^RTE^100^ replaced by QAQ (MyrUSH3 QAQ). The pair of oppositely charged residues was replaced by neutral glutamine, whereas the central residue was mutated to alanine. We used SPR to measure the reversible binding kinetics and the affinities to electrically neutral DOPC, negatively charged DOPC:DOPG (3:1), or DOPC:DOPG (2:1) SLBs obtained from liposome immobilization on phytosphingosine-derivatized sensor chips (XanTec) (Figures S5–S7 and Table S1).

**Figure 5:**
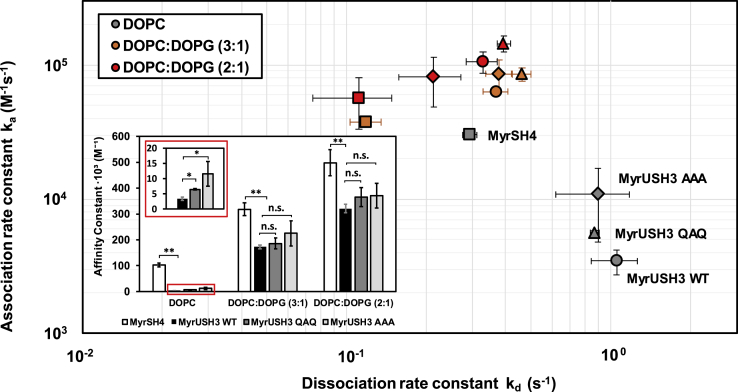
The Unique and SH3 Domains Modulate the Binding of MyrSH4 to Lipids SPR analysis of the binding of c-Src variants to immobilized DOPC (neutral) and DOPC:DOPG (3:1) and DOPC:DOPG (2:1) (negatively charged) liposomes. The main plot presents the association and dissociation rate constants, and the inset presents the affinity constant. The affinity constants with neutral lipids are also represented in an expanded scale. Data were fitted to a 1:1 Langmuir model (see Supplemental Information). Data are expressed as mean ± SD, n = 3. Significant differences in binding constants with respect to MyrUSH3 are indicated by asterisks (t test: *p < 0.05; **p < 0.01; n.s. not significant).

MyrSH4 showed two orders of magnitude higher affinity than the USH3 construct toward neutral SLBs (Figure 5). The AAA mutation in the UD or the QAQ mutation in the SH3 RT loop resulted in increased lipid-binding affinity caused by a faster association rate, suggesting a higher availability of the myristoyl group in the mutated USH3 forms. In contrast, dissociation rates were very similar, suggesting that the mutated sites do not directly interact with the lipid membrane. The lower dissociation rates of the isolated myristoylated SH4 peptide suggest that the neighbor UD and SH3 domain not only modulate the way the myristoylated SH4 domain is anchored to the membrane but also may reflect its higher tendency to form persistently bound oligomers (Le Roux et al., 2016a, Le Roux et al., 2016b).

These results are consistent with the NMR data indicating that the myristoyl group is interacting with the RT loop of the SH3 domain assisted by the ULBR in the UD.

With negatively charged lipids all the affinities increased owing to the additional electrostatic interaction with the positively charged SH4 domain, but the relative differences were reduced. A possible explanation is that the dissociation of the myristoyl group from the SH3 domain is enhanced by the electrostatic interaction of the strongly charged SH4 domain when it is close to the negatively charged membrane.

## Discussion

Protein myristoylation contributes to c-Src membrane anchoring together with a cluster of basic residues that interact with acidic phospholipids. Binding of c-Src to the membrane is mostly reversible, although evidence for clustering and irreversible binding of a small population of c-Src molecules has been reported (Le Roux et al., 2016a, Le Roux et al., 2016b, Smith et al., 2016, Owen et al., 2010). This is compatible with rapid exchange between bilayers of different cellular compartments by “hopping” through short-term cytosolic release of c-Src (Kasahara et al., 2007). Trafficking between plasma membrane and endosomes in the perinuclear region (Sandilands and Frame, 2008) may involve solubilizing proteins that recruit c-Src released from the membrane (Konitsiotis et al., 2017). Donepudi and Resh reported that approximately 30% of intracellular c-Src is not bound to membranes (Donepudi and Resh, 2008). This raises the question of possible interactions of the myristoyl group in the non-membrane-bound form of c-Src. The existence of internal myristoyl-binding sites may provide a modulating mechanism. A myristoyl-binding pocket is present in the C-lobe of the kinase domain of c-Abl and contributes to maintain it in the inactive state (Pluk et al., 2002). The presence of a similar binding pocket in c-Src has been suggested by Patwardhan and Resh (2010) on the basis of previous results by Cowand-Jacob et al. (2005) showing the interaction of free myristate with Tyr527-phosphorylated c-Src, although the binding site could not be identified.

Our results show that there is a myristoyl-binding site in the SH3 domain. The interaction of the myristoyl group with the SH3 domain restricts the availability of the fatty acid chain to bind to lipids and could similarly prevent its interaction with the kinase domain of another c-Src molecule.

Recently, N-terminal-bound myristoyl group has been suggested to mediate c-Src dimerization by interaction with the kinase domain (Spassov et al., 2018). Mutation of residues predicted to be part of the myristoyl-binding site in the kinase domain affected the observed dimers, giving support to the existence of a myristoyl-binding site also in this domain.

Thus the myristoyl group can interact with the same c-Src molecule to which it is bound (through the SH3 domain) or the kinase domain of a second c-Src protein. The two binding events may be linked. In fact, Spassov et al. showed that Y419 phosphorylation of the kinase domain, which changes its interaction with the SH3 domain, was required in *cis*, i.e., in the same molecule containing the myristoyl group for myristoyl-SH1-mediated dimerization to occur.

The AAA mutation prevents lipid binding by the UD in non-myristoylated constructs. However, this mutation results in an increase in the association rate of the myristoylated constructs with lipids. Thus the effect of the AAA mutation cannot be explained by changes in the direct lipid-binding capacity of the UD, but probably involves an indirect effect of the ULBR on the interaction of the myristoylated SH4 domain with the SH3 domain.

The AAA mutation in full-length c-Src has a dramatic effect on the invasive capacity of c-Src-dependent colorectal cancer cells. At the molecular level, myristoylation resulted in duplicated NMR signals only in the presence of the native ULBR sequence, suggesting that the native ULBR contributes to the interaction between the myristoyl group and the SH3 domain. A functional role of the ULBR is also suggested by the very different dynamics leading to distinct broadening of SH4 NMR signals of myristoylated constructs containing either the native ULBR sequence or the AAA mutation in the presence of liposomes.

Although our data do not provide any precise structural model, we can speculatively suggest that the ULBR contributes to modulating the balance between the lipid-bound and free forms of c-Src or the way by which MyrSH4 is anchored to the membrane while preserving the fuzzy intramolecular complex. This role of the UD would be in line with the notion that this intrinsically disordered region, exquisitely sensitive to the cellular environment and tunable, for example, by post-translational modifications or alternative splicing (Arbesú et al., 2018, Teixeira et al., 2018), could modulate the key events controlling the activation and localization of Src family kinases.

Our results have uncovered a myristoyl-binding site in the SH3 domain of c-Src and have shown that the fuzzy complex previously characterized in non-myristoylated proteins is retained in the myristoylated forms, either free or bound to lipid bilayers. From the structural point of view, it implies that the structured part of c-Src, including the SH3 domain, is located closer to the membrane surface than implied with earlier models in which the UD was considered a long spacer.

### Limitations of the Study

The interaction between the myristoyl group and the SH3 domain has been demonstrated *in vitro*. The interaction sites contributing to the fuzzy complex have been shown to be compatible with the known X-ray structure of full-length c-Src. As the myristoylated SH4 and SH3 domains are known to independently participate in other regulatory interactions, the known effects of preventing myristoylation, changing the acyl group, or mutating the SH3 domain do not provide additional insight. The disordered UD is not observed in X-ray structures. Direct observation of full-length myristoylated c-Src by NMR, *in vitro* or *in vivo,* is not yet technically possible but is the goal of our future research.

## Methods

All methods can be found in the accompanying Transparent Methods supplemental file.

## Supplemental Information

Document S1. Transparent Methods, Figures S1–S7, and Table S1
