# Supplementary material for: A Myristoyl-Binding Site in the SH3 Domain Modulates c-Src Membrane Anchoring
Source: iScience. 2019 Jan 14;12:194–203. doi: 10.1016/j.isci.2019.01.010 (PMC6354742; doi:10.1016/j.isci.2019.01.010)
Supplement: Document S1. Transparent Methods, Figures S1–S7, and Table S1 [file mmc1.pdf]

ISCI, Volume *12*

Supplemental Information

A Myristoyl-Binding Site in the SH3 Domain Modulates c-Src Membrane Anchoring

Anabel-Lise Le Roux, Irrem-Laareb Mohammad, Borja Mateos, Miguel Arbesú, Margarida Gairí, Farman Ali Khan, João M.C. Teixeira, and Miquel Pons

Supplementary information.

A

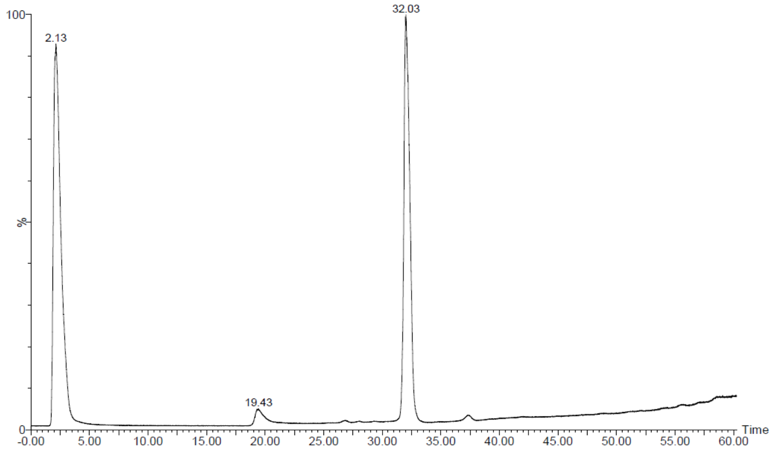

B

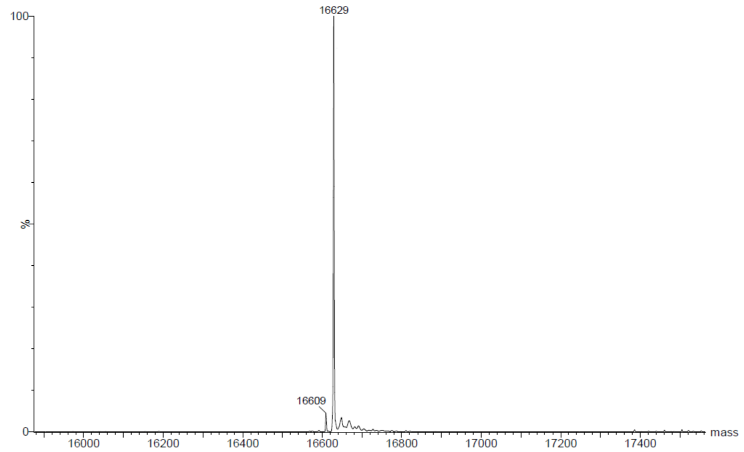

Supplementary Figure S1. MyrUSH3 characterization. Related to Figure 2. A) HPLC and B) MS analysis of MyrUSH3 WT. The observed molecular weight is in agreement with the theoretical molecular weight 16628 Da.

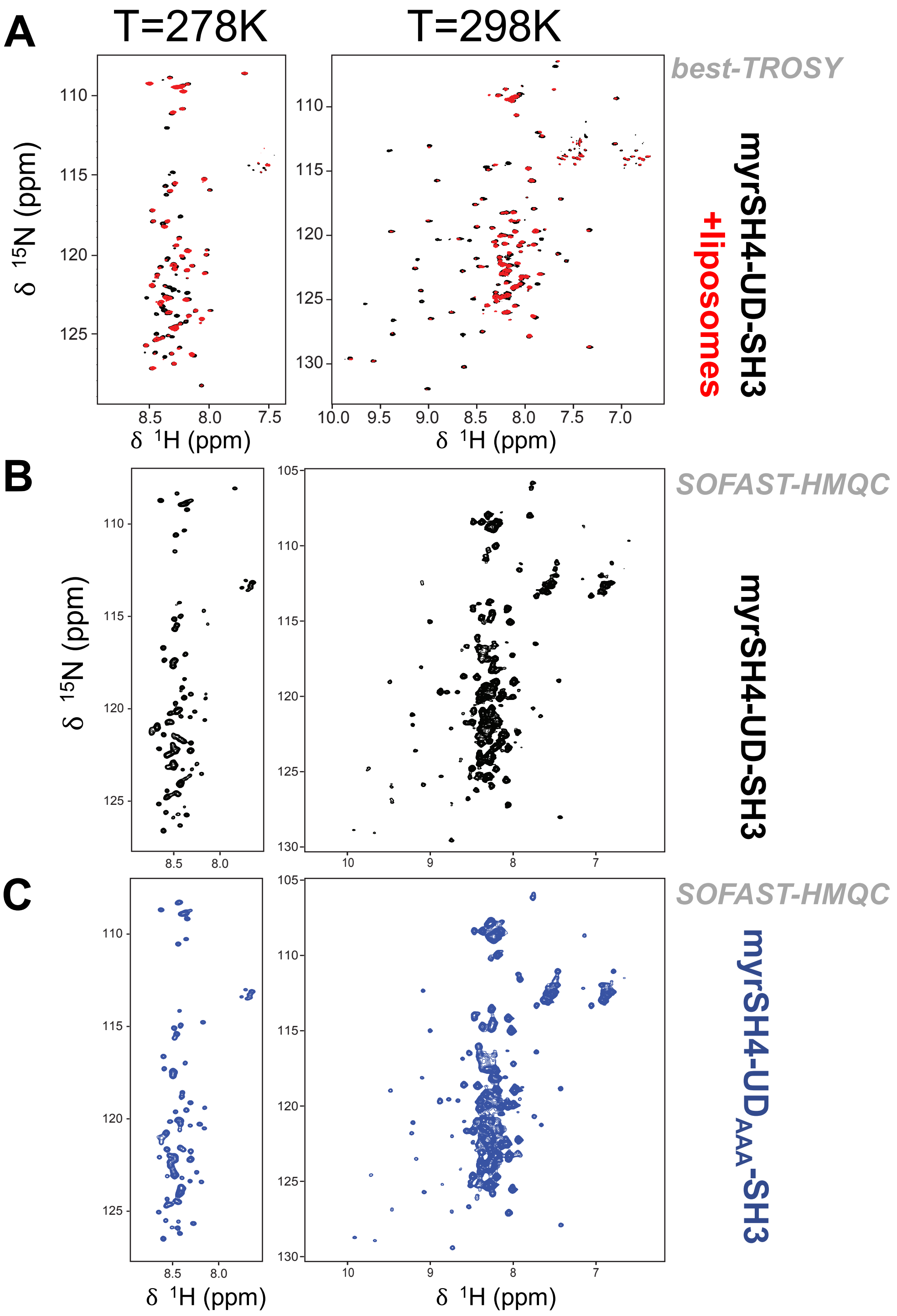

Supplementary Figure S2. Overview NMR spectra. Related to Figures 1-3 (A) ^{1}H-^{15}N bestTROSY at 278 K (left) and at 298 K (right) of native MyrUSH3 in the absence (black) or presence (red) of DOPC:DOPG (3:1) LUVs. (B) ^{1}H-^{15}N SOFAST-HMQC of MyrUSH3 WT at 278 K (left) and 298 K (right). (C) ^{1}H-^{15}N SOFAST-HMQC of MyrUSH3 AAA at 278 K (left) and 298 K (right).

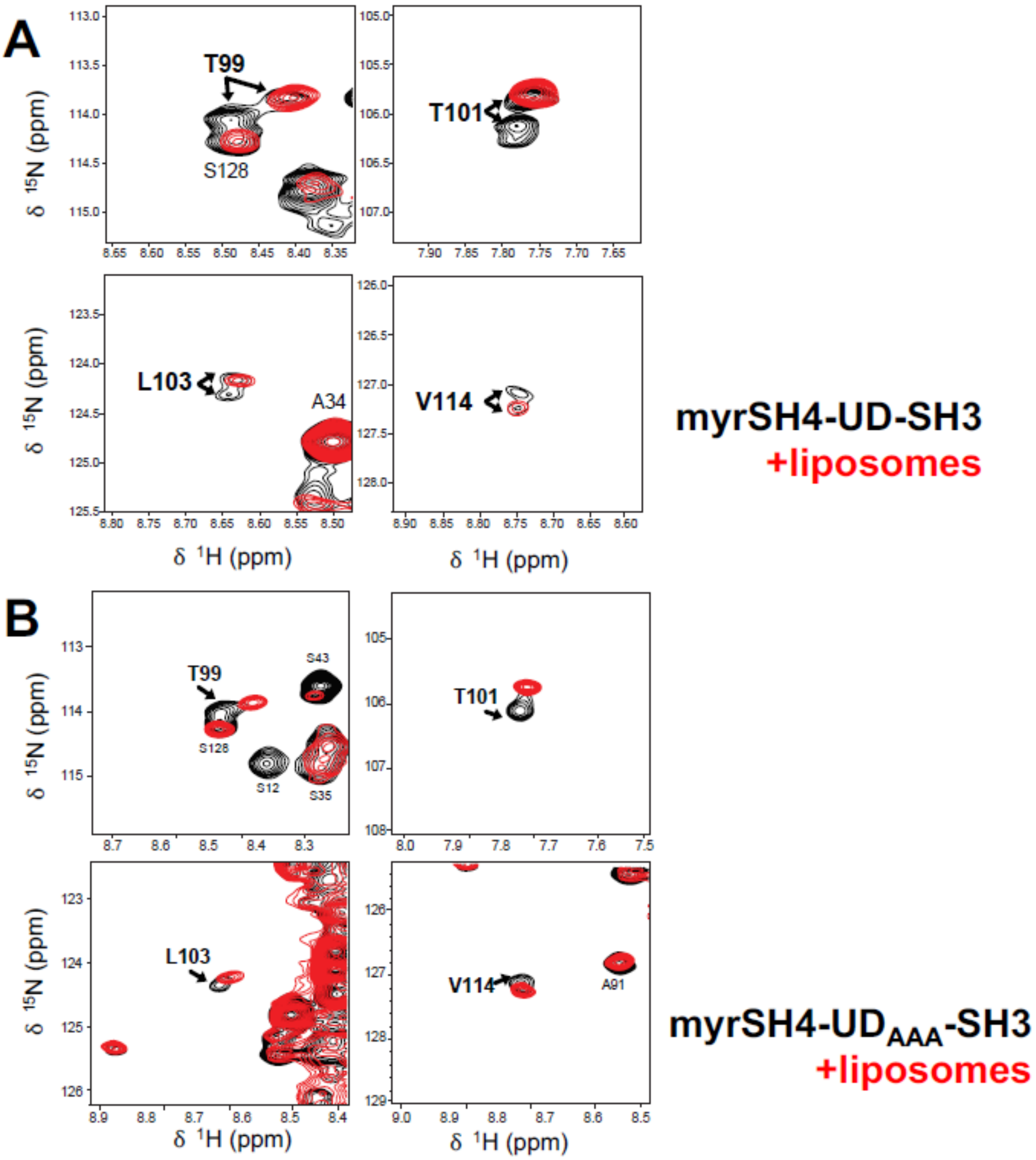

Supplementary Figure S3. Expanded NMR plots of duplicated signals. Related to Figure 2. Duplicated signals observed in the close environment of the RT-loop indicated in green in Figure 2 are shown here for the native MyrUSH3 (A) or MyrUS3 AAA (B). Only one of the duplicated signals remains observable in the presence of LUVs (red).

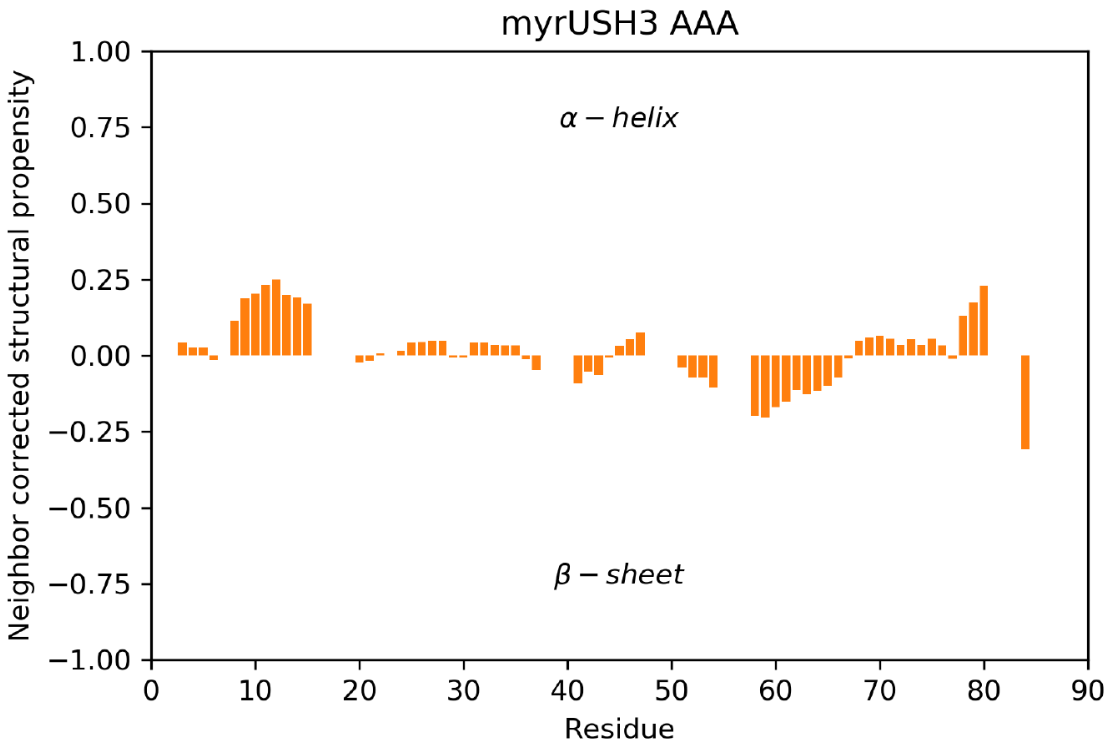

Supplementary Figure S4. Lack of secondary structure of AAA mutant. Related to Figure 2. Neighbor corrected structural propensity derived from the backbone chemical shifts of MyrUSH3 AAA at 278 K using ncSPC (Tamiola and Mulder, 2012). Structural propensities below 0.25 indicate that the introduction of three alanine residues in positions 63-65 in the AAA variant does not induce a significant ordering of the Unique domain.

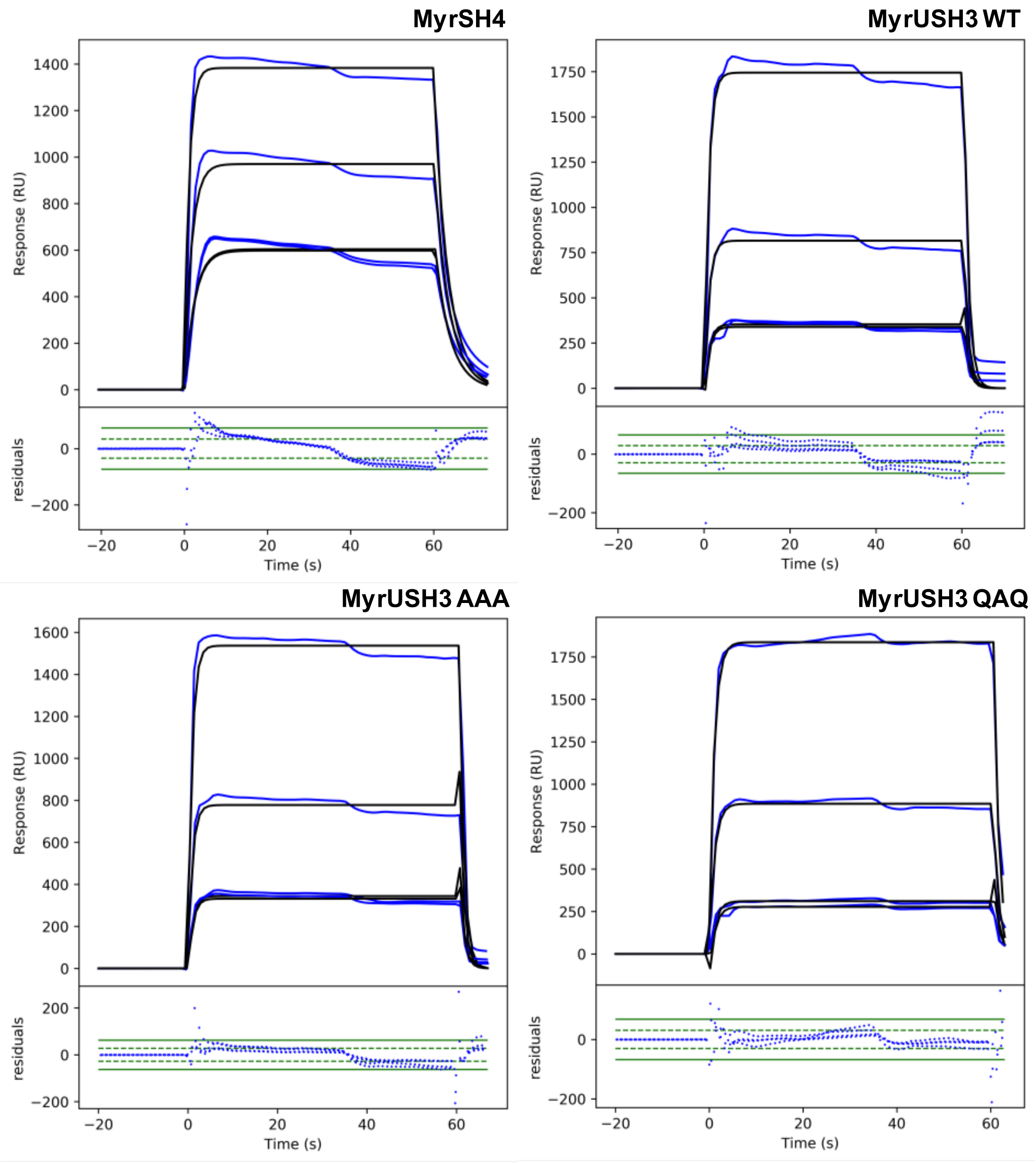

Supplementary Figure S5. SPR with neutral LUV. Related to Figure 5. SPR responses (blue curves) after 60 s association of the myristoylated c-Src variants to DOPC LUVs. Best fit with the 1:1 Langmuir model (black curves) analyzed using the Biacore T200 3.0 Evaluation. The residuals plot shows the accuracy of the fit. Result plots are one representative experiment of the triplicate data.

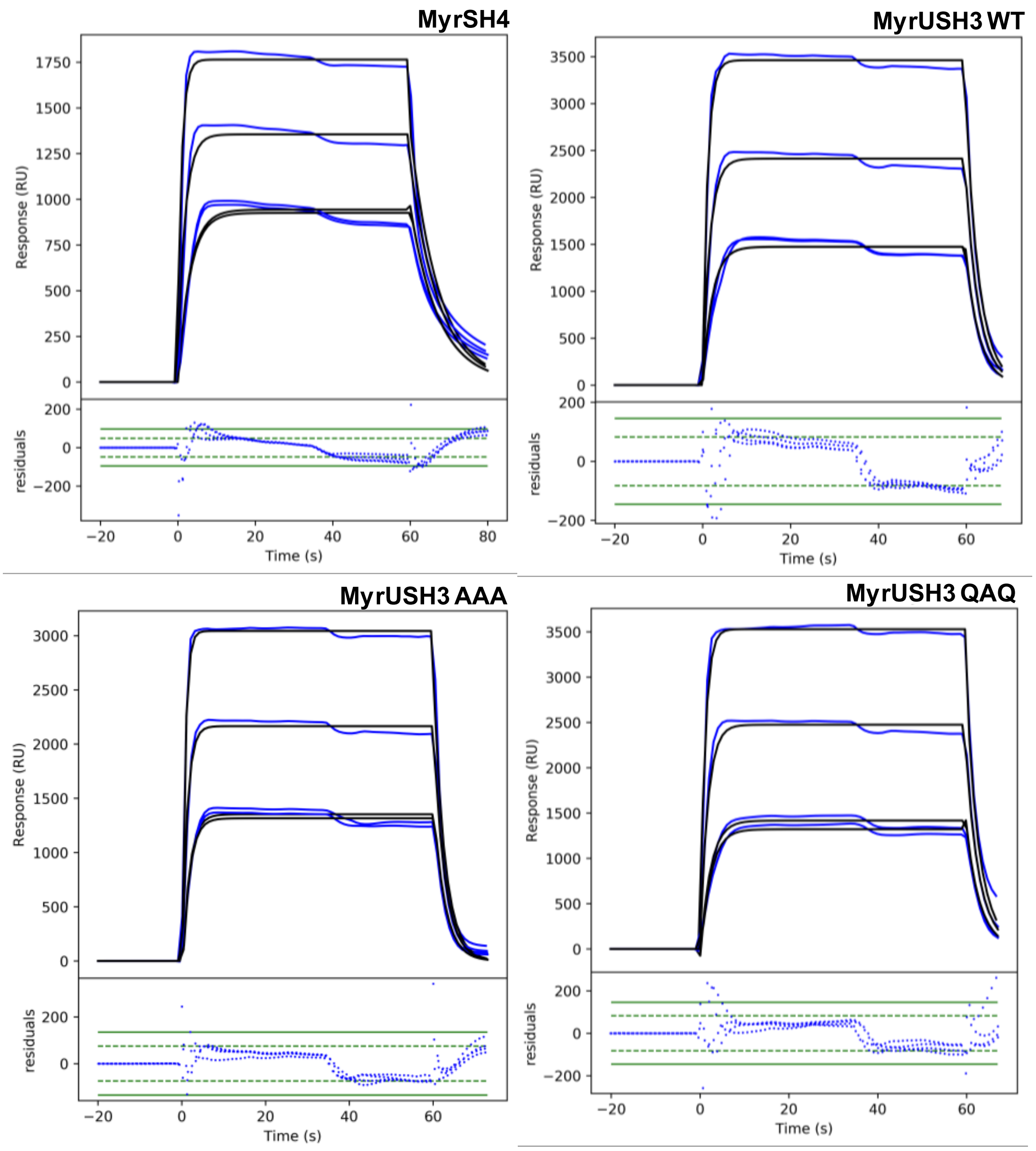

Supplementary Figure S6. SPR with 25% charged LUV. Related to Figure 5. SPR responses (blue curves) after 60 s association of the myristoylated c-Src variants to DOPC:DOPG (3:1) LUVs. Best fit with the 1:1 Langmuir model (black curves) analyzed using the Biacore T200 3.0 Evaluation. The residuals plot shows the accuracy of the fit. Result plots are one representative experiment of the triplicate data.

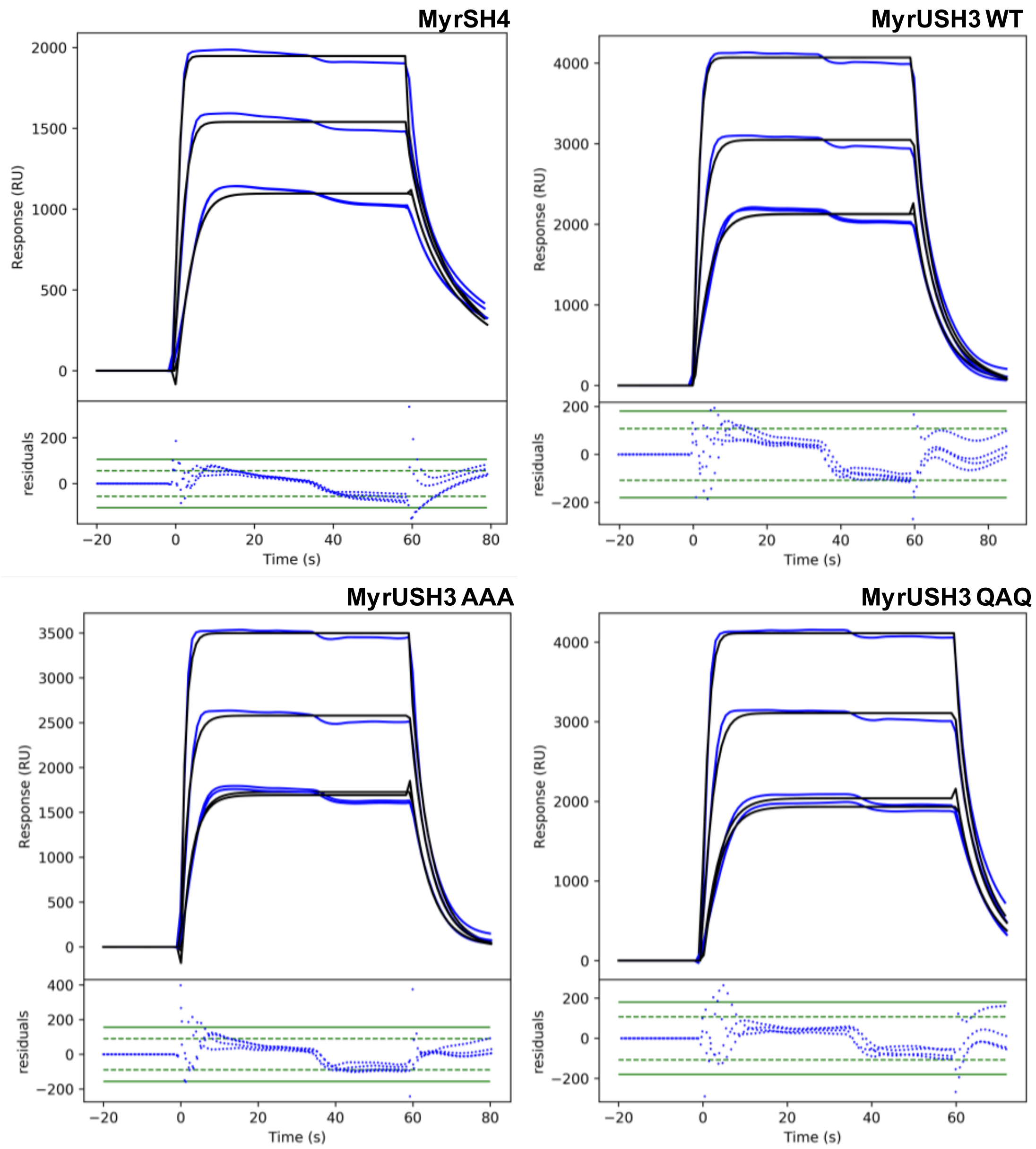

Supplementary Figure S7. SPR with 33% charged LUV. Related to Figure 5**.** SPR responses (blue curves) after 60s association of the myristoylated c-Src variants to DOPC:DOPG (2:1) LUVs. Best fit with the 1:1 Langmuir model (black curves) analyzed using the Biacore T200 3.0 Evaluation. The residuals plot shows the accuracy of the fit. Result plots are one representative experiment of the triplicate data.

Table S1. SPR derived constants. Related to Figure 5. Kinetic parameters obtained from the fitting of myristoylated c-Src variants to the different LUVs applying the 1:1 Langmuir model using the Biacore T200 3.0 Evaluation. These values are plotted in Figure 5.

LUVs	c-Src variant	k_a ($M^{-1}s^{-1}$)	k_d (s^{-1})	K_D (M)	K_A(M^{-1})
DOPC	MyrSH4	$3.24·10^4 ± 2.2·10^3$	0.293 ± 0.018	$9.76·10^{-6} ± 6.8·10^{-7}$	$1.03·10^5 ± 6.9·10^3$
	MyrUSH3 WT	$3.46·10^3 ± 7.3·10^2$	1.050 ± 0.210	$3.07·10^{-4} ± 5.2·10^{-5}$	$3.32·10^3 ± 5.2·10^2$
	MyrUSH3 QAQ	$5.6·10^3 ± 2.8·10^3$	0.870 ± 0.035	$1.56·10^{-4} ± 6.98·10^{-6}$	$6.44·10^3 ± 2.9·10^2$
	MyrUSH3 AAA	$1.09·10^4 ± 6.1·10^3$	0.896 ± 0.278	$9.27·10^{-5} ± 2.8·10^{-5}$	$1.16·10^4 ± 4.1·10^3$
DOPC:DOPG (3:1)	MyrSH4	$3.76·10^4 ± 2.36·10^3$	0.119 ± 0.016	$3.16·10^{-6} ± 2.51·10^{-7}$	$3.18·10^5 ± 2.47·10^4$
	MyrUSH3 WT	$6.33·10^4 ± 4.9·10^3$	0.368 ± 0.040	$5.80·10^{-6} ± 2.65·10^{-7}$	$1.73·10^5 ± 7.73·10^3$
	MyrUSH3 QAQ	$8.56·10^4 ± 9.7·10^3$	0.462 ± 0.036	$5.43·10^{-6} ± 5.9·10^{-7}$	$1.86·10^5 ± 2.1·10^4$
	MyrUSH3 AAA	$8.59·10^4 ± 2.34·10^4$	0.379 ± 0.042	$4.60·10^{-6} ± 1.04·10^{-6}$	$2.25·10^5 ± 4.93·10^4$
DOPC:DOPG (2:1)	MyrSH4	$5.64·10^4 ± 2.3·10^4$	0.111 ± 0.037	$2.02·10^{-6} ± 1.9·10^{-7}$	$4.98·10^5 ± 5.1·10^4$
	MyrUSH3 WT	$1.06·10^5 ± 1.9·10^4$	0.328 ± 0.044	$3.12·10^{-6} ± 1.6·10^{-7}$	$3.21·10^5 ± 1.6·10^4$
	MyrUSH3 QAQ	$1.44·10^5 ± 2.03·10^3$	0.394 ± 0.023	$2.75·10^{-6} ± 2.6·10^{-7}$	$3.65·10^5 ± 3.6·10^4$
	MyrUSH3 AAA	$8.09·10^4 ± 3.2·10^4$	0.214 ± 0.056	$2.73·10^{-6} ± 3.5·10^{-7}$	$3.70·10^5 ± 4.8·10^4$

Transparent methods

Myristoylated c-Src variants expression and purification

The myristoylated c-Src variants were obtained by the co-expression of the N-myristoyl transferase enzyme and the USH3 of c-Src substrate in a pETDuet-1 (Novagen) plasmid. The USH3 construct of c-Src, contains the SH4, Unique and SH3 domains, followed by a His_6 purification tag. The mutations were introduced using the QuickChange II XL Site Directed Mutagenesis Kit (Agilent).

Plasmids were transformed in *Escherichia coli* Rosetta^{TM} (DE3) pLysS (Novagen) and the bacteria cells were grown in Luria Broth (LB) medium supplemented with chloramphenicol (25 µg/mL) and ampicillin (100 µg/mL) at 37 ºC until an OD_{600nm} of ~0.6 was reached. Before induction with 1mM of isopropyl-β-D-thiogalactopyranoside (Nzytech), 6 g/L of glucose and a freshly prepared solution of myristic and palmitic acid (Sigma) (200 µM final concentration for each) and fatty acid free Bovine Serum Albumin (BSA) (Sigma) (600 µM final concentration), were added to the cell culture. The lipid solution was prepared by adding one equivalent of NaOH, heating at 65 ^oC and adjusting the final pH to 8. The protein expression was performed for 5 h at 28 ^oC. For ^{15}N-labeled protein the Marley method was used (Marley et al., 2001). After growing the cells in LB medium as previously described, cultures were harvested by centrifugation at 1000 g for 30 min and resuspended in M9 medium containing 1 g/L ^{15}N NH_4Cl (Cambridge Isotope Laboratories). Before inducing the expression, 3 g/L of glucose and a freshly prepared solution of myristic and palmitic acid (Sigma) at 50 µM final concentration for each, with fatty acid free BSA (Sigma) at 600 µM final concentration, were added to the cell culture. The expression was performed as above.

Cells were harvested at 4000 rpm for 20 min and resuspended in lysis buffer (20 mM Tris·HCl, 300 mM NaCl, 10 mM Imidazole, pH 8) supplemented with

Protein Inhibitor Cocktail (Sigma) and 1 mM Phenylmethanesulfonyl fluoride (PMSF) (Sigma). Cells were sonicated on ice and centrifuged at 25000 rpm for 45 min. The myristoylated protein was extracted from the pellet using lysis buffer supplemented with 1 % Triton X100 (Sigma). Subsequently, Ni-NTA affinity chromatography was performed using a 1 mL-Ni-NTA cartridge (GE Healthcare). The protein was eluted with lysis buffer supplemented with 400 mM imidazole and 0.02 % Triton X100 (Sigma). The final purification step consisted of a size exclusion chromatography in a Superdex 75 26/60 (GE Healthcare), in phosphate buffer (50 mM NaP, 150 mM NaCl, 0.2 mM EDTA, pH 7.5). For NMR, samples buffer was exchanged to 50 mM NaP pH 7.0 using a P10 column (GE Healthcare). The purity of the protein was established by HPLC in a BioSuite pPhenyl 1000RPC 2.0 x 75 mm; 10 µm column coupled to mass spectrometry, confirming the absence of lauroylated protein. The protein was concentrated either using Vivaspin 20, 5 kDa MWCO concentrators (Sigma Aldrich) or dialyzing the sample against double distilled water with a subsequent lyophilization and resuspension with buffer. Myristoylated SH4 (MyrSH4) was synthesized by SynPeptide Co., Ltd (Shangai, China).

Preparation of Large Unilamellar vesicles (LUVs)

1,2-dioleoyl-*sn*-glycero-3-phosphocoline (DOPC) (TebuBio) and 1,2-dioleoyl-sn-glycero-3-phospho(1'-rac-glycerol) (sodium salt) (DOPG) (Sigma) were dissolved in chloroform. Three lipid compositions were used: DOPC, DOPC:DOPG (3:1) and DOPC:DOPG (2:1). The organic solvent was evaporated under a nitrogen stream. The lipid films were rehydrated with phosphate buffer (50 mM NaP, 150 mM NaCl, 0.2 mM EDTA, pH7.5) with vortexing. LUVs were prepared by extrusion using a Mini-extruder (Avanti Polar Lipids). The lipid suspension was extruded 15 times through a 100 nm-polycarbonate filter. The mean diameter of the LUVs was verified by Dynamic Light Scattering (Zetasizer Nanoseries S, Malvern instruments). LUVs were used within two days to avoid lipid oxidation.

NMR Experiments

NMR experiments were performed in a Bruker 600 MHz Avance III spectrometer equipped with a TCI Cryoprobe. ^{1}H-^{15}N best-TROSY (Solyom et al. 2013) or ^{1}H,^{15}N SOFAST-HMQC experiments (Schanda et al. 2005) were measured at 278 K or 298 K. The samples contained 0.075-0.2 mM protein concentration in 50 mM NaP, pH 7.0 with 10 % D_2O. NMR data was processed with nmrPipe (Delaglio et al. 1995) and analyzed with Sparky (Lee et al. 2015), ccpNmr (Vranken et al. 2005). Plots were generated with Farseer-NMR (Teixeira et al. 2018) and R. CSP were computed as $CSP(ppm) = \sqrt{\frac{1}{2}[\delta_H^2 + (0.2 \cdot \delta_N^2)^2]}$. The threshold line in the plots represent the mean value of the lowest 10% CSP plus five standard deviations.

The assignment of the MyrUSH3 AAA construct was carried out in a ^{13}C, ^{15}N uniformly labeled sample using an automatic assignment strategy (Jaravine & Orekhov, 2006) based on co-processing of HNCO, HNcaCO HNCA, HNcoCA, HNCACB and HNcoCACB spectra acquired using non-uniform sampling in the

NMR facility of the University of Goteborg (Sweden). The assignments have been deposited in BMRB:27708.

Surface Plasmon Resonance binding assays

SPR experiments were performed in a Biacore T200 instrument (GE Healthcare). The temperature was set to 25 C in all the experiments. The 2D-carboxylmethyldextran sensor chip (Xantec) was used. All the channels, except for the reference, were modified by the covalent attachment of phytosphingosine (TebuBio) to allow the capture of LUVs. An amine-coupling procedure was performed with 1 mM of phytosphingosine in acetate buffer pH 6.7. Phosphate buffer (50 mM NaP, 150 mM NaCl, 0.2 mM EDTA, pH 7.5) was used as running buffer for all the experiments. DOPC, DOPC:DOPG (3:1) and DOPC:DOPG (2:1) LUVs at 1 mM concentration were coated over the three different channels (maintaining this order to avoid anionic lipid migration towards the neutral LUVs through the flow cells) by a 20 s injection at 10 µL/min. The reference cell and possible uncovered surface in the LUVs channels were blocked with 1 mg/ml of BSA at 50 µL/min for 20 s. To minimize mass transport effects, the myristoylated c-Src variants were injected at 50 µL/min. Protein concentration ranged from 1.5 µM to 20 µM. For each c-Src construct three protein concentrations were injected with one in duplicate and in randomized order. The protein was allowed to associate for 60 s while the dissociation lasted 120 s. Triplicate experiments were performed for each c-Src variant. The surface was regenerated with two pulses (30 s at 100 µL/min) of Isopropanol:50 mM NaOH (2:3) solution followed by a 20 mM CHAPS pulse. Each binding experiment was started with freshly captured LUVs. LUV coating was reproducible, obtaining an average value of 7958.8 ±172.6 RU for DOPC LUVs, 6093.3 ±153.4 RU for DOPC: DOPG (3:1) and 5613.5 ±145.3 RU for DOPC: DOPC (2:1) (± standard deviation).

The myristoylated c-Src variants binding to LUVs were analyzed using the Biacore T200 3.0 Evaluation software (GE Healthcare). The protein concentration was corrected by subtracting any degradation present using SDS PAGE. All data was double referenced (reference channel and baseline subtraction) and globally fitted using a simple 1:1 Langmuir model. The kinetics of binding and dissociation were measured at three concentrations with one of them duplicated. The curves were fitted globally. Each experiment was repeated three times (Figures S5-S7 and Table S1).

Supplementary references

Delaglio F., Grzesiek, S., Vuister, G. W., Zhu, G., Pfeifer, J., .and Bax, A. (1995). NMRPipe: a multidi,mensional spectral processing system based on UNIX pipes, J. Biomol. NMR. 6, 277-293.

Jaravine, V.A. and Orekhov, V.Y. (2006). Targeted Acquisition for Real-Time NMR Spectroscopy. J. Am. Chem. Soc. 128, 13421-13426

Lee, W., Tonelli, M., Markley, J.L. (2015). NMRFAM-SPARKY: enhanced software for biomolecular NMR spectroscopy. Bioinformatics. 31, 1325-7.

Marley, J., Lu, M., Bracken (2001). A method for efficient isotopic labeling of recombinant proteins. J. Biomol. NMR, 20, 71-75.

Schanda, P., Kupce, E., Brutscher, B. (2005). SOFAST-HMQC experiments for recording two-dimensional heteronuclear correlation spectra of proteins within a few seconds. J Biomol NMR. 33, 199-211.

C. Solyom, Z., Schwarten, M.,Geist, L., Konrat, R., Willbold, D., Brutscher B. (2013) BEST-TROSY experiments for time-efficient sequential resonance assignment of large disordered proteins. J Biomol NMR. 55, 311-21.

Teixeira, J.M.C., Skinner, S.P., Arbesú, M., Breeze, A.L. Pons, M. (2018). Farseer-NMR: automatic treatment, analysis and plotting of large, multi-variable NMR data. J Biomol NMR. 2018 doi: 10.1007/s10858-018-0182-5.

Vranken, W.F., Boucher, W., Stevens, T.J., Fogh, R.H., Pajon, A., Llinas, M., Ulrich, E.L., Markley, J.L., Ionides, J. and Laue, E.D. (2005). The CCPN data model for NMR spectroscopy: development of a software pipeline. Proteins. 59, 687-96.
